# Laparoscopic versus Open Hepatectomy with or without Synchronous Colectomy for Colorectal Liver Metastasis: A Meta-Analysis

**DOI:** 10.1371/journal.pone.0087461

**Published:** 2014-01-29

**Authors:** MingTian Wei, YaZhou He, JiaRong Wang, Nan Chen, ZongGuang Zhou, ZiQiang Wang

**Affiliations:** 1 Department of Gastrointestinal Surgery, West China Hospital, Sichuan University, Chengdu, Sichuan Province, China; 2 West China Medical School of Sichuan University, Chengdu, Sichuan Province, China; National Cancer Center, Japan

## Abstract

**Background:**

To compare short-term and long-term results of colorectal patients undergoing laparoscopic and open hepatectomy. Moreover, outcomes of laparoscopic versus open procedures for simultaneous primary colorectal tumor and liver metastasis resection were compared.

**Methods:**

A systematic search was conducted in the PubMed and EmBase databases (until Oct. 22. 2013) with no limits. Bibliographic citation management software (EndNote X6) was used for extracted literature management. Quality assessment was performed according to a modification of the Newcastle-Ottawa Scale. The data were analyzed using Review Manager (Version 5.1), and sensitivity analysis was performed by sequentially omitting each study.

**Results:**

Finally, 14 studies, including a total of 975 CLM (colorectal liver metastasis) patients, compared laparoscopic with open hepatectomy. 3 studies of them, including a total of 107 CLM patients, compared laparoscopic with open procedures for synchronous hepatectomy and colectomy. Laparoscopic hepatectomy was associated with a significantly less blood loss, shorter hospitalization time, and less operative transfusion rate. In addition, lower hospital morbidity rate (OR = 0.57, 95%CI:0.42–0.78, P = 0.0005) and better R0 resection (OR = 2.44, 95%CI:1.21–4.94, P = 0.01) were observed in laparoscopic hepatectomy. For long-term outcomes, there were no significant differences between two surgical procedures on recurrence and overall survival. In comparison of synchronous hepatectomy and colectomy, laparoscopic procedure displayed shorter hospitalization (MD = −3.40, 95%CI:−4.37–2.44, P<0.00001) than open procedure. Other outcomes, including surgical time, estimated blood loss, hospital morbidity, and overall survival did not differ significantly in the comparison.

**Conclusions:**

Laparoscopic hepatectomy with or without synchronous colectomy are acceptable for selective CLM patients. We suggest standard inclusion criteria of CLM patients be formulated.

## Introduction

Nowadays, colorectal liver metastasis (CLM) is gaining wide population from multi-disciplinary doctors including gastroenterologists, oncologists, and hepatic doctors for its increasing incidence and poor prognosis. 20%–25% of colorectal cancer patients present with simultaneous liver metastasis at the time of diagnosis and a few patients are evaluated with primary tumor and liver metastasis resectable synchronously [1]. Although the use of chemotherapy regimen has been certified favorable outcomes, liver resection is still recommended as the optional treatment for CLM patients.

Recently, with the advantages of earlier recovery, shorter hospitalization, and lower morbidity, laparoscopic hepatectomy for CLM has been performed in few specialized centers [2]–[4]. Owing to the complex technique and patient selection, there is no study of large scale of patients reporting the short- and long-term results for laparoscopic hepatectomy in CLM, especially compared with open procedure. Moreover, the optimal time for liver resection still remains controversial [5], and whether the staged resection of primary colorectal cancer and liver metastasis will benefit to 1-stage resection of both tumors is known.

To address these issues, our team conducted the following meta-analysis to compare short-term and long-term results of CLM patients undergoing laparoscopic and open hepatectomy. In addition, outcomes of laparoscopic versus open procedures for simultaneous primary colorectal tumor and liver metastasis resection were compared.

## Methods

This meta-analysis was conducted following the Cochrane Handbook for Systematic Reviews of Interventions 5.1.0 (updated March 2011) to ensure data quality (http://www.cochrane.org/training/cochrane-handbook).

### Search for studies

A systematic search was conducted in the PubMed and EmBase databases (until 22 October 2013) with no limits. Our search strategies were listed as follows. PubMed database: ((((((laparoscop*) OR celioscop*)) OR laparoscopy[MeSH Terms])) AND ((liver metasta*) OR hepatic metasta*)) AND ((colorectal neoplasms[MeSH Terms]) OR ((((((colon cancer) OR colon carcinoma) OR rectal cancer) OR rectal carcinoma) OR colorectal cancer) OR colorectal carcinoma)). Embase database: (((colon cancer or colon carcinoma or rectal cancer or rectal carcinoma or colorectal cancer or colorectal carcinoma).all fields. or (colon cancer or colon carcinoma or rectal cancer or rectal carcinoma or colorectal cancer or colorectal carcinoma).key words.) AND ((Liver metasta* or hepatic metasta*).all fields. or (Liver metasta* or hepatic metasta*).key words.)) AND ((laparoscop* or celioscop*).all fields. or (laparoscop* or celioscop*).key words.). Moreover, previously published reviews on the topic of interest were obtained and checked. We traced the reference list of relevant articles and used Google Scholar to find potential studies.

### Study selection

Study designs included random controlled studies (RCTs), clinical controlled studies (CCTs), cohort studies, and case-control studies.

The inclusion criteria were as follows: (1) diagnosis of colorectal liver metastasis in adult patients, (2) the surgical procedure compares laparoscopic and open regimens, (3) the studies provides short- or long-term outcomes, and (4) available data for each surgical regimen. We excluded studies including (1) animal model was used for comprising two surgical regimens, (2) the patients in the study were diagnosis of mixed disease, such as benign and malignant liver tumors, (3) just one surgical regimen (laparoscopic or open) was reported, (4) other minimally invasive surgery such as radiofrequency ablation was compared, (5) the studies were reviews, letters, abstracts and editorial material, and (6) studies lacking available data.

We imported the search results into bibliographic citation management software (EndNote X6). Two reviewers independently screened studies by reading titles and abstracts to roughly identify potential reports. The full texts of articles for all references identified as matching the inclusion criteria were obtained. Inclusion criteria were applied to the full texts. Disagreement was resolved through discussion and asking for advice from corresponding authors.

### Data extraction and quality assessment

Two reviewers independently extracted data from eligible studies, and any disagreement was adjudicated by discussion or consulting the corresponding author. Baseline information included first author, published year, surgical approaches, study design, region, numbers of cases, and mean age among other parameters. The following outcome data were extracted: (1) short-term outcome: surgical related outcome, oncological outcome (R0 resection, and tumor-free margin), complications (bile leakage) and (2) long-term outcome: recurrence and overall survival.

For non-random controlled studies, a modification of the Newcastle-Ottawa Scale (NOS) was used as an assessment tool for selection, comparability and outcome assessment [6]. Five main factors from clinical risk score were considerate: positive node of primary tumor, disease-free interval, number of liver metastases, presence of liver tumor, CEA level [7]. In order to evaluate selection as accurately as possible, age, sex, ASA score, and pre- and post-operative chemotherapies were also taken into consideration. Out of a total of six stars, studies valued more than four stars were recognized as being moderate to high quality.

### Outcome definition and statistical analysis

Perioperative mortality was defined as 30-day hospital death. Hospitalization time included total hospital time and postoperative time. Local recurrence included hepatic only, extrahepatic only, and both hepatic and extrahepatic which were observed till the end of follow-up. Complications contained hepatic and extrahepatic complications. Subgroup analysis was set in the studies which reported comparison of laparoscopic and open simultaneous primary tumor and liver metastasis.

The data were analyzed using Review Manager (Version 5.1). Odds ratios (ORs) or risk differences (RDs) along with 95% confidence intervals (CI) were used for analyzing dichotomous data, and mean differences (MDs) along with 95%CI for continuous data. For survival analysis, we extracted data from survival curve referring to method reported in previous study, and hazard ratio was used for quantitative analysis [8]. To assess the variation across studies, heterogeneity was measured with the I^2^ index and P value. Based on method reported by DerSimonian and Laird [9], substantial significance was set when P<0.10 and a random effect model was used [9], [10]. Otherwise, a fixed-effect model was considered. Standard deviation (SD) was estimated by a formula when only a range was reported: Estimate SD = Range/4 (15<n<70); Range/6 (n>70), and median was approximately equal to mean [11]. The value of P<0.05 was considered to indicate statistical significance. With regard to outcomes when significant heterogeneity existed across studies, sensitivity analysis was performed by sequentially omitting each study to test the influence of individual study on pooled data.

## Results

### Characteristics of pooled studies

**Figure 1:**
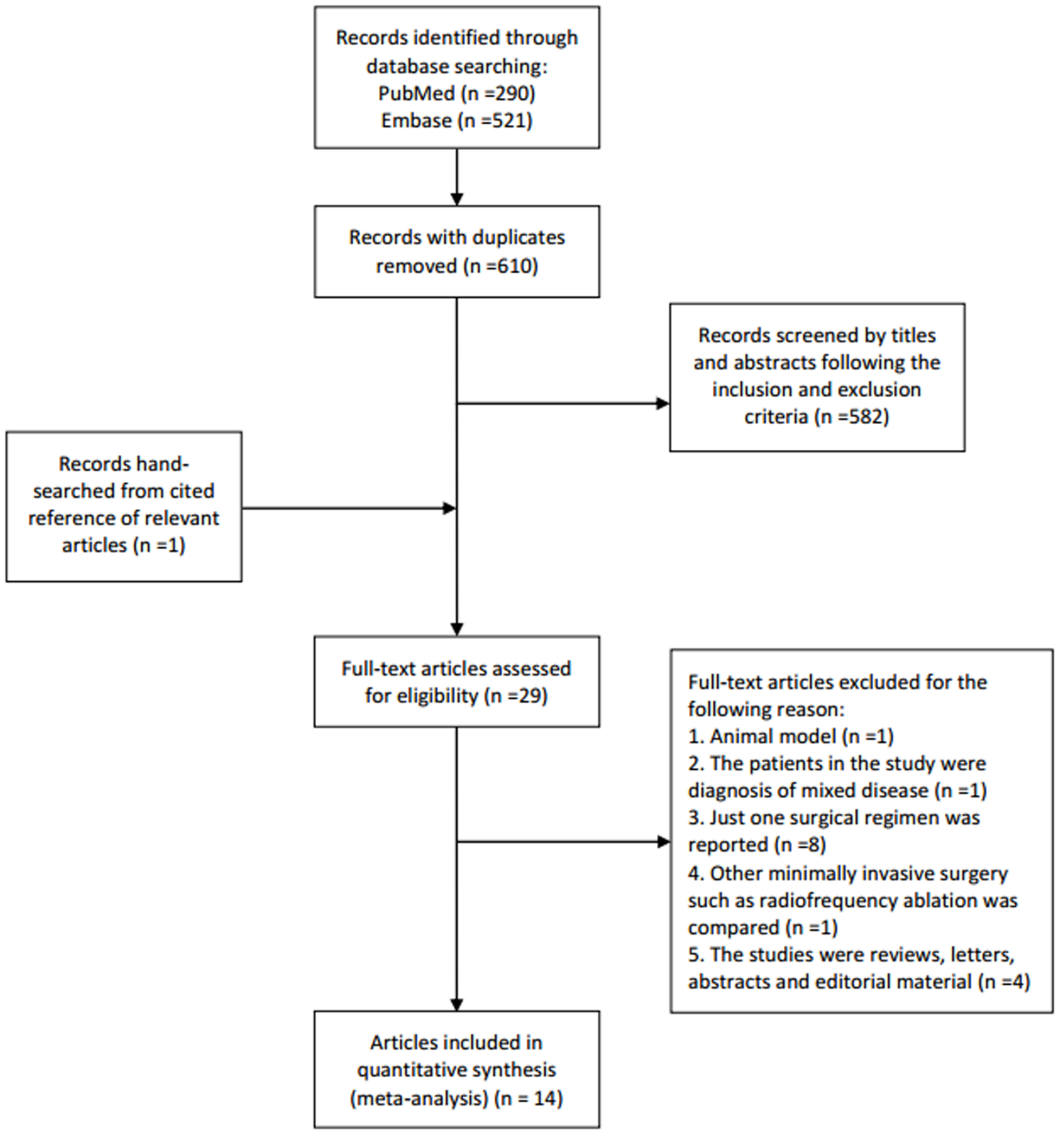
Flow diagram of meta-analysis study selection process.

A total of 610 potential abstracts were identified after removing out duplication in the primary search of the electronic databases. A flow diagram of the detailed selection process is shown in Figure 1. Finally, 14 studies including a total of 975 CLM patients compared the outcomes between laparoscopic hepatectomy and open hepatectomy [12]–[25]. Among them, 3 studies (107 patients) compared laparoscopic hepatectomy and open hepatectomy synchronously combined with primary colorectal tumor resection [15], [19], [20]. All the patients in the 3 studies underwent synchronous hepatectomy and colectomy simultaneous resection. For the other 11 studies, 5 studies reported all patients underwent single liver resection [12], [16], [18], [21], [23]. The residual 6 studies reported a portion of patients underwent liver resection and the other patients underwent synchronous hepatectomy and colectomy in each study [13], [14], [17], [22], [24], [25]. Table 1 offers the baseline characteristics of all studies.

**Table 1:** Basic characteristics of all pooled studies in the meta-analysis.

First author year (ref.)	No. of patients		Follow-up (median)		Type of resection	Country	Conversion (%)
	Laparoscopic	Open	Laparoscopic	Open			
Abu Hilal et al, 2010 [12]	50	85	22	28	liver	United Kingdom	12
Cannon et al, 2012 [13]	35	138	>60^†^	>60^†^	liver/simul	United States	NA
Castaing et al, 2009 [14]	60	60	30	33	liver/simul	France	10
Chen et al, 2011 [15]	23	18	45.3	45.3	simul	China	0
Cheung et al, 2012 [16]	20	40	NA	NA	liver	Hong Kong	NA
Doughtie et al, 2013 [17]	8	76	23	23	liver/simul	United States	0
Guerron et al, 2013 [18]	40	40	16	16	liver	United States	5
Hu et al, 2012 [19]	13	13	16–81^‡^	16–81^‡^	simul	China	0
Huh et al, 2011 [20]	20	20	27.4	27.4	simul	Korea	0
Inoue et al, 2013 [21]	23	24	NA	NA	liver	Japan	4.3
Iwahashi et al, 2013 [22]	21	21	>60^†^	>60^†^	liver/simul	Japan	0
Mala et al, 2002 [23]	13	14	NA	NA	liver	Norway	0
Qiu et al, 2013 [24]	30	30	NA	NA	liver/simul	China	6.6
Topal et al, 2012 [25]	20	20	43.4	43.4	liver/simul	Belgium	NA

NA, not available; No., number; ^†^: upper ends of follow-up range; ^‡^: range of follow-up; liver: all the patients in the study only underwent liver resection; liver/simul: a portion of patients in the study underwent liver resection and the other patients underwent synchronous hepatectomy and colectomy; simul: all the patients in the study underwent synchronous hepatectomy and colectomy; All study design was case-control.

### Quality judgments of studies

Owing to the specificity and ethics, surgeons can hardly randomly allocate CML patients into laparoscopic or open liver resection. Rigorous literature research also shows few experimental trails focus on the topic we are concerning. Thus, the included 14 researches were retrospectively case-control studies. Table 2 lists the evaluation stars of each study followed by the modified NOS. In the selection of patients, all studies reviewed consecutive CLM patients, however, laparoscopic surgery was manipulated in selected patients who were suitable, and the patients accepting open surgery were selectively matched. All included studies were comparable. Overall, all studies were evaluated as being moderate to high quality.

**Table 2:** Quality assessment of studies in the meta-analysis based on modified NOS judgment.

First author, year	Selection		Comparability	Outcome assessment		Quality judgment
	1	2	3	4	5	
Abu Hilal et al, 2010	★	–	★	★	★	★★★★
Cannon et al, 2012	★	★	★★	★	★	★★★★★★
Castaing et al, 2009	★	★	★★	★	★	★★★★★★
Chen et al, 2011	★	–	★	★	★	★★★★
Cheung et al, 2012	★	★	★	★	★	★★★★★
Doughtie et al, 2013	★	–	★★	★	★	★★★★★
Guerron et al, 2013	★	★	★	★	★	★★★★★
Hu et al, 2012	★	★	★	★	★	★★★★★
Huh et al, 2011	★	–	★★	★	★	★★★★★
Inoue et al, 2013	★	–	★★	★	★	★★★★★
Iwahashi et al, 2013	★	★	★★	★	★	★★★★★★
Mala et al, 2002	★	–	★★	★	★	★★★★★
Qiu et al, 2013	★	★	★★	★	★	★★★★★★
Topal et al, 2012	★	★	★★	★	★	★★★★★★

**Selection**: 1. Is the subject definition adequate or described? (if yes, one star); 2. Was the subject representative of the total population? (one star, if truly or obviously; no stars if subjects were selected group or not described). **Comparability**: 3. Did the study have no differences between laparoscopic and liver resection for CLM? Five main factors were considerate: positive node of primary tumor, disease-free interval, number of liver metastases, presence of liver tumor, CEA level. Other four factors: age, sex, ASA score, and pre- and postoperative chemotherapy were comparative (if yes, two stars; one star if there were no other differences between the two groups even if one or more of these five characteristics was not reported; no star was assigned if the two groups differed). **Outcome assessment**: 4. Clearly defined outcome of interest (if yes, one star); 5. Adequacy of follow-up (one star if less than 20% of CLM patients lost to follow-up, otherwise no stars).

### Comparison in all types of hepatectomy

With respect to operative related outcomes, four endpoints including surgical time, estimated blood loss, operative transfusion, and hospitalization time were taken into analysis. Surgical time was assessed with no significant difference between laparoscopic and open liver resection for CLM (MD = 5.01, 95%CI:−8.92–18.94, P = 0.48). In contrast, less blood loss, shorter hospitalization time and less operative transfusion rate in laparoscopic liver resection were all highly significant (P<0.00001–0.0008). Moreover, perioperative mortality did not differ in both surgical approaches (Table 3).

**Table 3:** Pooled outcomes of laparoscopic versus procedures in all studies.

Outcomes	Number of studies	Participants		MD/OR/RD [95% CI]	P value for effect size	Test of heterogeneity		Analysis model
		Laparoscopic	Open			I^2^ (%)	P value	
surgical time	12	333	385	5.10 [−8.92, 18.94]^a^	0.48	60	0.004	Random
estimated blood loss	12	308	463	−182.87 [−263.50, −102.25]^a^	<0.0001	90	<0.00001	Random
hospitalization time	11	312	364	−3.39 [−4.29, −2.48]^a^	<0.00001	66	0.001	Random
operative transfusion	9	274	434	0.41 [0.24, 0.69]^b^	0.0008	7	0.37	Fixed
perioperative mortality	11	297	422	−0.01 [−0.03, 0.01]^c^	0.58	0	1.00	Fixed

MD, mean difference; OR, odds ratio; RD, risk difference; CI, confidence intervals; ^a^, MD; ^b^, OR; ^c^, RD.

**Figure 2:**
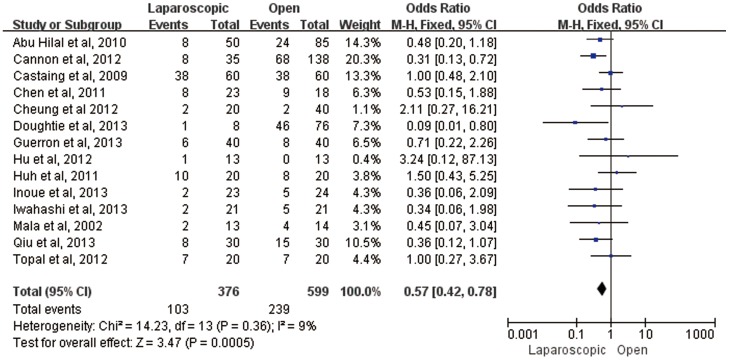
Forest plot of morbidity in all included studies.

**Figure 3:**
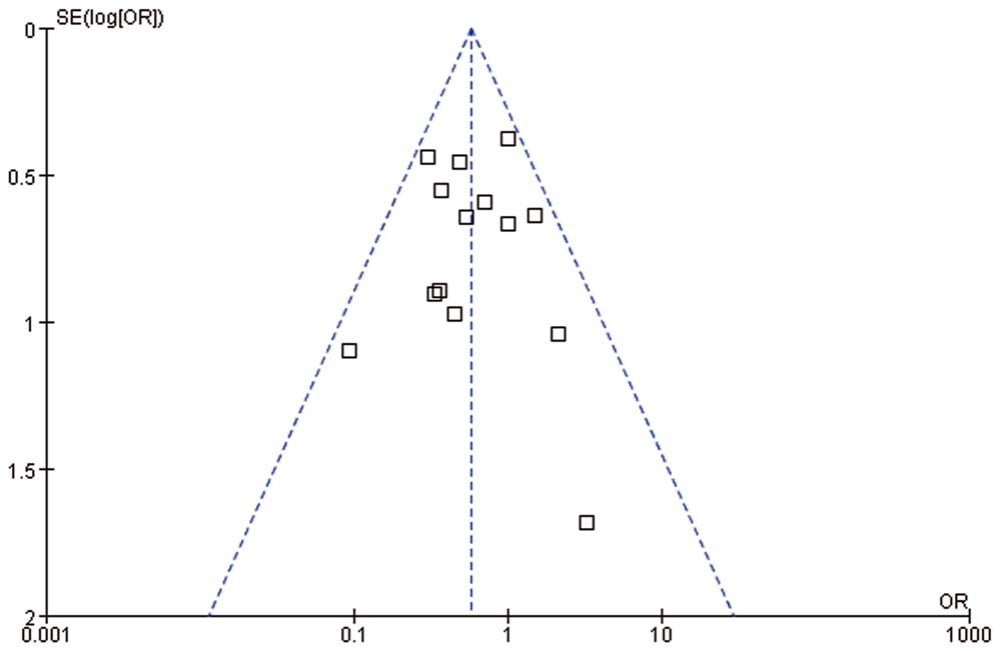
Funnel plot of morbidity in all included studies.

In the quantitative analysis of hospital morbidity, laparoscopic liver resection is significantly superior to open liver resection (OR = 0.57, 95%CI:0.42–0.78, P = 0.0005) (Figure 2). Specially, one main complication of bile leakage did not differ significantly between laparoscopic and open liver resection for CLM (OR = 0.81, 95%CI:0.30–2.20, P = 0.69). Funnel plot of morbidity showed no obvious evidence of publication bias (Figure 3).

**Figure 4:**
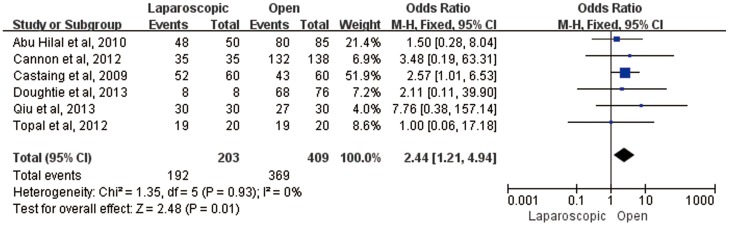
Forest plot of oncological results on R0 resection.

**Figure 5:**
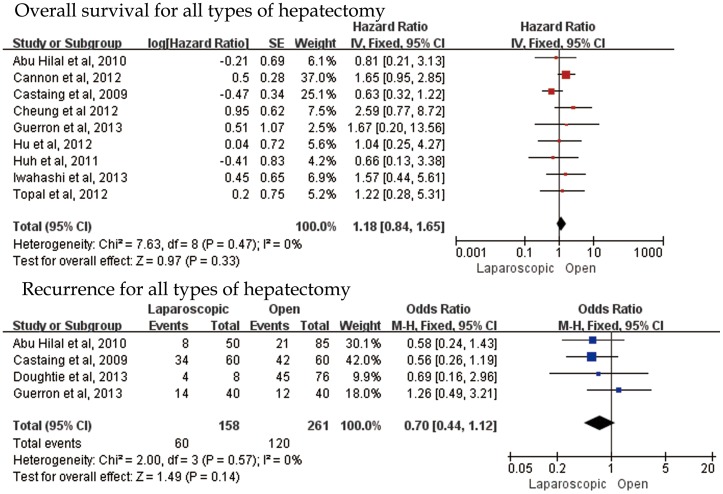
Forest plot of long-term results on overall survival and recurrence in all types of hepatectomy.

Considering oncologic outcomes, laparoscopic liver resection acquire shorter tumor-free margin compared with open liver resection for CLM (MD = −0.79, 95%CI:−1.54–0.05, P = 0.04). However, significant R0 resection benefit was found in laparoscopic liver resection in comparison of two surgical approaches (OR = 2.44, 95%CI:1.21–4.94, P = 0.01) (Figure 4).In regards to overall survival in all types of hepatectomy which was the crucial endpoint, 9 studies reported available Kaplan-Meier survival curves, and laparoscopic procedure did not bring about significant benefits (HR = 1.18, 95%CI:0.84–1.65, P = 0.33). Concerning local recurrence, we pooled hepatic only, extrahepatic only, and both hepatic and extrahepatic recurrence from reported studies. 6 studies were hit into our analysis and fixed-effect was used. Among the 6 studies, 2 reported no local recurrence [13], [23]. Our outcome was prone to a lower recurrence rate in laparoscopic approach compared with open approach (OR = 0.70, 95%CI:0.44–1.12, P = 0.14) (Figure 5).

### Comparison in type of synchronous hepatectomy and colectomy

**Figure 6:**
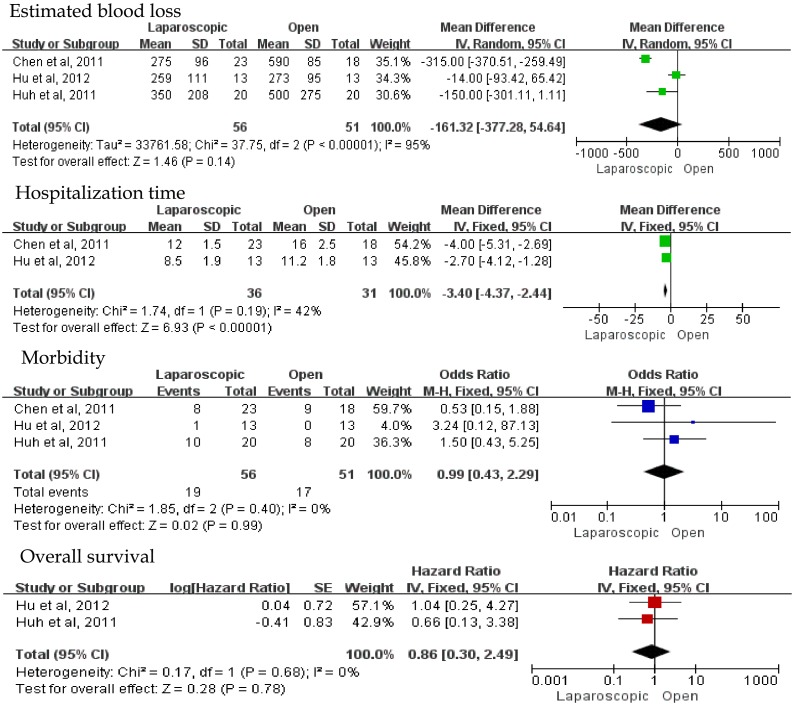
Forest plot of merged results in type of hepatectomy and synchronous colectomy.

Due to the limited number of researches, there were only 3 studies comparing two surgical approaches for patients with synchronous hepatectomy and colectomy. The results demonstrated parallel blood loss, shorter hospitalization time, and similar surgical time in laparoscopic approach compared with open approach (Figure 6). In addition, estimated outcome of hospital morbidity in this group did not show any significant difference between laparoscopic and open liver combined with simultaneous colorectal resection (HR = 0.99, 95%CI:0.43–2.29, P = 0.99) (Figure 6).

In terms of long-term result, 2 studies were pooled in the analysis and no significant difference of overall survival was observed between laparoscopic and open procedures for patients with synchronous hepatectomy and colectomy (HR = 0.86, 95%CI: 0.30–2.49, P = 0.78). In addition, the other study reported 1-, 3- and 5-year survival rates in laparoscopic approach compared with open approach (82.6% vs. 77.8%, 43.5% vs. 38.9%, and 8.6% vs. 0, respectively) (P>0.05).

### Heterogeneity

High heterogeneity was detected concerning surgical time (I^2^ = 60%, P = 0.004), estimated blood loss (I^2^ = 90%, P<0.0001), and hospitalization time (I^2^ = 66%, P = 0.001) in all types of hepatectomy. And then sensitivity analysis was conducted by omitting each single study. There were no changes of outcomes compared with primary outcomes. In addition, high heterogeneity was also detected for surgical time (I^2^ = 84%, P = 0.002) in type of synchronous hepatectomy and colectomy and the sensitivity did not alter the outcome.

## Discussion

In spite of the first description of laparoscopic hepatectomy for liver tumor in 1990s, it does not develop rapidly due to concern of intraoperative hemorrhage, port-site recurrence, as well as complex laparoscopic technique [26], [27]. Subsequent improvement of instruments and technique make laparoscopic liver resection actively performed. And recent initial experiences have reported the feasibility, safety, and efficacy of laparoscopic liver resection for CLM [28]–[30]. However, the studies are retrospectively observational or of small patients number. There is no convincing evidence on the safety and efficacy of laparoscopic hepatectomy for CLM, let alone synchronously laparoscopic hepatectomy and colectomy.

Our goal of this meta-analysis was to evaluate the efficacy as well as safety and feasibility of laparoscopic liver resection for CLM. In the present study, we compared short- and long-term results of laparoscopic versus open hepatectomy for colorectal metastasis. Moreover, laparoscopic versus open hepatectomy and colectomy synchronously was also compared. Our results renew a latest meta-analysis on laparoscopic hepatectomy which was limited by inadequately pooled data [31]. The comparison in included studies was well matched through nine aspects (Table 2) which provided relatively similar baseline. Our pooled outcome provides a convincing evidence for the popularization of laparoscopic liver resection for selective CLM. However, caution should be taken care to explain the pooled results because of the limitations of our study.

With regard to oncological outcomes, R0 resection is the goal of all surgeries which is defined conventionally as tumor-free margin of more than 1 centimeter. As endoscopic procedures and promising techniques develop, intraoperative laparoscopic ultrasonography has been applied for decision of appropriate hepatoectomy, which makes adequate resection of tumor-free margin feasible [29], [32]. And also, some institutions prefer intraoperative frozen biopsy to identify potential tumor-positive margin. However, oncological R0 resection is closely related with location and number or size of metastasis, especially when liver metastasis locates near hilum or over 10 centimeters size. For those patients, compulsive laparoscopic resection often causes failure oncological resection and poor prognosis, thus those often account for contraindications of laparoscopic hepatectomy for CLM. Finally, our estimated outcome favors an obvious R0 benefit from laparoscopic liver resection compared with open procedure in selective patients.

To our knowledge, overall survival is closely related with oncological results [33], [34]. Our analysis demonstrates considerable profile compared with open resection, though laparoscopic hepatectomy failures to increase significant overall survival rate. We compare our estimation with previous studies. Survey from large European institutions has reported a range 46%–64% of 5-year overall survival in laparoscopic hepatectomy [35], [36]. Nguyen and his colleague also declares laparoscopic hepatectomy will bring about a 50% of 5-year overall survival in United States [37]. Besides, all included studies show both surgical approaches are connected with equivalent overall survival which is consistent with that in the estimated outcome.

Hemorrhage or surgical blood loss caused by laparoscopic hepatectomy is a potential concern for hepatic doctors, especially some unskillful laparoscopic surgeons. However, our analysis on estimated surgical blood loss and operative transfusion rate shows a benefit of laparoscopic resection than open resection. Similar finding is also reported by multiple studies [38], [39]. In accordance with other researches, other short-term benefits of hospitalization time and hospital morbidity are demonstrated in laparoscopic resection compared with open resection [23]. Indeed, laparoscopic surgery has been proved with the advantages of less blood loss, shorter hospitalization, better recovery, and lower morbidity in various aspects [40], [41], so does laparoscopic hepatectomy.

Another concern our meta-analysis focus on is comparison of laparoscopic and open hepatectomy and colectomy synchronously for CLM. As we know, laparoscopic colectomy is widely accepted as favorable treatment for CRC, and laparoscopic liver resection for primary liver tumor is routinely recommended. So combination of laparoscopic synchronously hepatectomy and colectomy for CLM is proposed in recent years. And some studies have demonstrated simultaneous resection of liver metastasis and primary colorectal tumor feasible and safe for CLM, with the merits of avoiding reoperation for secondary resection, and preventing advanced lesion progress [42]–[44]. However, the development of laparoscopic technique into hepatectomy makes the optional choice of surgical approach controversial. Although some preliminary studies have reported their experience dealing with laparoscopic or open simultaneously resection for CLM, our subgroup analysis hits 3 studies comparing both surgical approaches. Pooled outcome demonstrates laparoscopic hepatectomy and colectomy synchronously did not increase morbidity and overall survival which was accordance with reported researches [42], [45].

Proper patient selection for laparoscopic and open hepatectomy for CLM remains a major topic the surgeons focus on. There is no standard inclusion criteria for appropriate surgical option which is often decided after a multi-disciplinary consultation. And laparoscopic technical difficulty and complexity of metastatic lesions limit comparisons of two surgical approaches to observational studies. In order to reduce potential selection bias, we extract matched comparative studies into our current analysis as accurately as possible. And the pooled outcomes provide powerful evidence of prior laparoscopic hepatectomy compared with open hepatectomy for CLM. However, we suggest systemic train of laparoscopic skills of high order be planed for hepatic doctors, moreover, a selection standard of CLM patients for laparoscopic surgery should be formulated.

There are some limitations in this meta-analysis. (1) Some indirect data acquisition methods were used, such as when dealing with the standard deviation from range. (2) Relatively high heterogeneity among studies was estimated for surgical related outcomes, especially in surgical time, estimated blood loss, and hospitalization. (3) Our included studies were all observational which may decrease the power of our outcome.

## Conclusion

In summary, we identified better short-term results in laparoscopic hepatectomy for CLM patients, and equivalent recurrence and overall survival were observed in both laparoscopic and open procedures. Similar results were also demonstrated in comparison of synchronous hepatectomy and colectomy. So, we conclude that laparoscopic hepatectomy with or without synchronous colectomy are acceptable for selective CLM patients. We suggest standard inclusion criteria of CLM patients be formulated.

## Supporting Information

Checklist S1 PRISMA checklist.
(DOC)

## References

[1] Van CE, Nordlinger B, Adam R, Kohne C, Pozzo C, et al. (2006) Towards a pan-European consensus on the treatment of patients with colorectal liver metastases. Eur J Cancer 42: : 2212–2221. Epub 2006 Aug 2210. 10.1016/j.ejca.2006.04.012 16904315

[2] Akiyoshi T, Kuroyanagi H, Saiura A, Fujimoto Y, Koga R, et al (2009) Simultaneous resection of colorectal cancer and synchronous liver metastases: initial experience of laparoscopy for colorectal cancer resection. Dig Surg 26: 471–475. 20068319 10.1159/000237109

[3] Bretagnol F, Hatwell C, Farges O, Alves A, Belghiti J, et al.. (2008) Benefit of laparoscopy for rectal resection in patients operated simultaneously for synchronous liver metastases: preliminary experience. Surgery 144: : 436–441. doi 410.1016/j.surg.2008.1004.1014. Epub 2008 Jul 1010. 10.1016/j.surg.2008.04.014 18707042

[4] Leung KL, Lee JF, Yiu RY, Ng SS, Li JC (2006) Simultaneous laparoscopic resection of rectal cancer and liver metastasis. J Laparoendosc Adv Surg Tech A 16: 486–488. 17004874 10.1089/lap.2006.16.486

[5] Pathak S, Sarno G, Nunes Q, Poston G (2010) Synchronous resection for colorectal liver metastases: the future. Eur J Surg Oncol 36: : 1044–1046. doi 1010.1016/j.ejso.2010.1008.1137. Epub 2010 Sep 1015. 10.1016/j.ejso.2010.08.137 20833502

[6] Athanasiou T, Al-Ruzzeh S, Kumar P, Crossman M, Amrani M, et al (2004) Off-pump myocardial revascularization is associated with less incidence of stroke in elderly patients. Ann Thorac Surg 77: 745–753. 14759484 10.1016/j.athoracsur.2003.07.002

[7] Fong Y, Fortner J, Sun R, Brennan M, Blumgart L (1999) Clinical score for predicting recurrence after hepatic resection for metastatic colorectal cancer: analysis of 1001 consecutive cases. Ann Surg 230: 309–318 discussion 318–321. 10493478 10.1097/00000658-199909000-00004 PMC1420876

[8] Parmar M, Torri V, Stewart L (1998) Extracting summary statistics to perform meta-analyses of the published literature for survival endpoints. Stat Med 17: 2815–2834. 9921604 10.1002/(sici)1097-0258(19981230)17:24<2815::aid-sim110>3.0.co;2-8

[9] DerSimonian R, Laird N (1986) Meta-analysis in clinical trials. Control Clin Trials 7: 177–188. 3802833 10.1016/0197-2456(86)90046-2

[10] Higgins JP, Thompson SG, Deeks JJ, Altman DG (2003) Measuring inconsistency in meta-analyses. BMJ 327: 557–560. 12958120 10.1136/bmj.327.7414.557 PMC192859

[11] Hozo S, Djulbegovic B, Hozo I (2005) Estimating the mean and variance from the median, range, and the size of a sample. BMC Med Res Methodol 5: 13. 15840177 10.1186/1471-2288-5-13 PMC1097734

[12] Abu Hilal M, Underwood T, Zuccaro M, Primrose J, Pearce N (2010) Short- and medium-term results of totally laparoscopic resection for colorectal liver metastases. Br J Surg 97: 927–933. 20474003 10.1002/bjs.7034

[13] Cannon RM, Scoggins CR, Callender GG, McMasters KM, Martin RC 2nd (2012) Laparoscopic versus open resection of hepatic colorectal metastases. Surgery 152: 567–573 discussion 573–564. 22943842 10.1016/j.surg.2012.07.013

[14] Castaing D, Vibert E, Ricca L, Azoulay D, Adam R, et al (2009) Oncologic results of laparoscopic versus open hepatectomy for colorectal liver metastases in two specialized centers. Ann Surg 250: 849–855. 19801934 10.1097/SLA.0b013e3181bcaf63

[15] Chen KY, Xiang GA, Wang HN, Xiao FL (2011) Simultaneous laparoscopic excision for rectal carcinoma and synchronous hepatic metastasis. Chin Med J (Engl) 124: 2990–2992. 22040541

[16] Cheung T, Poon R, Yuen W, Chok K, Tsang S, et al (2012) Outcome of laparoscopic versus open hepatectomy for colorectal liver metastases. ANZ J Surg 4: 1445–2197. 10.1111/j.1445-2197.2012.06270.x 23035809

[17] Doughtie CA, Egger ME, Cannon RM, Martin RC, McMasters KM, et al (2013) Laparoscopic hepatectomy is a safe and effective approach for resecting large colorectal liver metastases. Am Surg 79: 566–571. 23711264

[18] Guerron A, Aliyev S, Agcaoglu O, Aksoy E, Taskin H, et al.. (2013) Laparoscopic versus open resection of colorectal liver metastasis. Surg Endosc 27: : 1138–1143. doi 1110.1007/s00464-00012-02563-00462. Epub 02012 Oct 00410. 10.1007/s00464-012-2563-2 23052537

[19] Hu MG, Ou-yang CG, Zhao GD, Xu DB, Liu R (2012) Outcomes of open versus laparoscopic procedure for synchronous radical resection of liver metastatic colorectal cancer: a comparative study. Surg Laparosc Endosc Percutan Tech 22: 364–369. 22874690 10.1097/SLE.0b013e31825af6b2

[20] Huh JW, Koh YS, Kim HR, Cho CK, Kim YJ (2011) Comparison of laparoscopic and open colorectal resections for patients undergoing simultaneous R0 resection for liver metastases. Surg Endosc 25: 193–198. 20549242 10.1007/s00464-010-1158-z

[21] Inoue Y, Hayashi M, Tanaka R, Komeda K, Hirokawa F, et al (2013) Short-term results of laparoscopic versus open liver resection for liver metastasis from colorectal cancer: a comparative study. Am Surg 79: 495–501. 23635585

[22] Iwahashi S, Shimada M, Utsunomiya T, Imura S, Morine Y, et al.. (2013) Laparoscopic hepatic resection for metastatic liver tumor of colorectal cancer: comparative analysis of short- and long-term results. Surg Endosc. 10.1007/s00464-013-3165-3 23996337

[23] Mala T, Edwin B, Gladhaug I, Fosse E, Soreide O, et al (2002) A comparative study of the short-term outcome following open and laparoscopic liver resection of colorectal metastases. Surg Endosc 16: 1059–1063. 12165823 10.1007/s00464-001-9176-5

[24] Qiu J, Chen S, Pankaj P, Wu H (2013) Laparoscopic hepatectomy for hepatic colorectal metastases – a retrospective comparative cohort analysis and literature review. PLoS One 8: : e60153. doi 60110.61371/journal.pone.0060153. Epub 0062013 Mar 0060121. 10.1371/journal.pone.0060153 PMC3605322 23555908

[25] Topal H, Tiek J, Aerts R, Topal B (2012) Outcome of laparoscopic major liver resection for colorectal metastases. Surg Endosc 26: 2451–2455. 22358126 10.1007/s00464-012-2209-4

[26] Johnstone P, Rohde D, Swartz S, Fetter J, Wexner S (1996) Port site recurrences after laparoscopic and thoracoscopic procedures in malignancy. J Clin Oncol 14: 1950–1956. 8656265 10.1200/JCO.1996.14.6.1950

[27] Paolucci V, Schaeff B, Schneider M, Gutt C (1999) Tumor seeding following laparoscopy: international survey. World J Surg 23: 989–995 discussion 996–987. 10512937 10.1007/s002689900613

[28] O'Rourke N, Shaw I, Nathanson L, Martin I, Fielding G (2004) Laparoscopic resection of hepatic colorectal metastases. HPB (Oxford) 6: : 230–235. doi 210.1080/13651820410023978. 10.1080/13651820410023978 PMC2020681 18333080

[29] Gigot J, Glineur D, Santiago AJ, Goergen M, Ceuterick M, et al (2002) Laparoscopic liver resection for malignant liver tumors: preliminary results of a multicenter European study. Ann Surg 236: 90–97. 12131090 10.1097/00000658-200207000-00014 PMC1422553

[30] Vibert E, Perniceni T, Levard H, Denet C, Shahri N, et al (2006) Laparoscopic liver resection. Br J Surg 93: 67–72. 16273531 10.1002/bjs.5150

[31] Zhou Y, Xiao Y, Wu L, Li B, Li H (2013) Laparoscopic liver resection as a safe and efficacious alternative to open resection for colorectal liver metastasis: a meta-analysis. BMC Surg 13: 44. 24083369 10.1186/1471-2482-13-44 PMC3849970

[32] Uchiyama K, Ueno M, Ozawa S, Kiriyama S, Shigekawa Y, et al.. (2010) Combined use of contrast-enhanced intraoperative ultrasonography and a fluorescence navigation system for identifying hepatic metastases. World J Surg 34: : 2953–2959. doi 2910.1007/s00268-00010-00764-00261. 10.1007/s00268-010-0764-1 20734045

[33] Abu HM, Di FF, Abu SM, Pearce N (2012) Oncological efficiency analysis of laparoscopic liver resection for primary and metastatic cancer: a single-center UK experience. Arch Surg 147: : 42–48. doi 10.1001/archsurg.2011.1856. 10.1001/archsurg.2011.856 22250111

[34] Feroci F, Baraghini M, Lenzi E, Garzi A, Vannucchi A, et al.. (2013) Laparoscopic surgery improves postoperative outcomes in high-risk patients with colorectal cancer. Surg Endosc 27: : 1130-1137. doi 1110.1007/s00464-00012-02559-y. Epub 02012 Oct 00466. 10.1007/s00464-012-2559-y 23052534

[35] Sasaki A, Nitta H, Otsuka K, Takahara T, Nishizuka S, et al.. (2009) Ten-year experience of totally laparoscopic liver resection in a single institution. Br J Surg 96: : 274–279. doi 210.1002/bjs.6472. 10.1002/bjs.6472 19224518

[36] Kazaryan A, Pavlik MI, Rosseland A, Rosok B, Mala T, et al.. (2010) Laparoscopic liver resection for malignant and benign lesions: ten-year Norwegian single-center experience. Arch Surg 145: : 34–40. doi 10.1001/archsurg.2009.1229. 10.1001/archsurg.2009.229 20083752

[37] Nguyen K, Laurent A, Dagher I, Geller D, Steel J, et al.. (2009) Minimally invasive liver resection for metastatic colorectal cancer: a multi-institutional, international report of safety, feasibility, and early outcomes. Ann Surg 250: : 842–848. doi 810.1097/SLA.1090b1013e3181bc1789c. 10.1097/SLA.0b013e3181bc789c 19806058

[38] Lesurtel M, Cherqui D, Laurent A, Tayar C, Fagniez P (2003) Laparoscopic versus open left lateral hepatic lobectomy: a case-control study. J Am Coll Surg 196: 236–242. 12595052 10.1016/S1072-7515(02)01622-8

[39] Abu Hilal M, Underwood T, Zuccaro M, Primrose J, Pearce N (2010) 1 Short- and medium-term results of totally laparoscopic resection for colorectal liver metastases. Br J Surg 97: 927–933. 20474003 10.1002/bjs.7034

[40] Pham T, Perry K, Dolan J, Schipper P, Sukumar M, et al.. (2010) Comparison of perioperative outcomes after combined thoracoscopic-laparoscopic esophagectomy and open Ivor-Lewis esophagectomy. Am J Surg 199: : 594–598. doi 510.1016/j.amjsurg.2010.1001.1005. 10.1016/j.amjsurg.2010.01.005 20466101

[41] Karakousis G, Singer S, Zheng J, Gonen M, Coit D, et al.. (2011) Laparoscopic versus open gastric resections for primary gastrointestinal stromal tumors (GISTs): a size-matched comparison. Ann Surg Oncol 18: : 1599–1605. doi 1510.1245/s10434-10010-11517-y. Epub 12011 Jan 10435. 10.1245/s10434-010-1517-y PMC4986692 21207158

[42] Weber J, Bachellier P, Oussoultzoglou E, Jaeck D (2003) Simultaneous resection of colorectal primary tumour and synchronous liver metastases. Br J Surg 90: 956–962. 12905548 10.1002/bjs.4132

[43] Tanaka K, Shimada H, Matsuo K, Nagano Y, Endo I, et al (2004) Outcome after simultaneous colorectal and hepatic resection for colorectal cancer with synchronous metastases. Surgery 136: 650–659. 15349115 10.1016/j.surg.2004.02.012

[44] Martin R, Paty P, Fong Y, Grace A, Cohen A, et al (2003) Simultaneous liver and colorectal resections are safe for synchronous colorectal liver metastasis. J Am Coll Surg 197: 233–241 discussion 241–232. 12892803 10.1016/S1072-7515(03)00390-9

[45] Thelen A, Jonas S, Benckert C, Spinelli A, Lopez-Hanninen E, et al. (2007) Simultaneous versus staged liver resection of synchronous liver metastases from colorectal cancer. Int J Colorectal Dis 22: : 1269–1276. Epub 2007 Feb 1221. 10.1007/s00384-007-0286-y 17318552

